# Modeling the Conductivity and Diffusion Permeability of a Track-Etched Membrane Taking into Account a Loose Layer

**DOI:** 10.3390/membranes12121283

**Published:** 2022-12-19

**Authors:** Vladlen S. Nichka, Semyon A. Mareev, Pavel Yu. Apel, Konstantin G. Sabbatovskiy, Vladimir D. Sobolev, Victor V. Nikonenko

**Affiliations:** 1 Physical Chemistry Department, Membrane Institute, Kuban State University, Krasnodar 350040, Russia; 2 Joint Institute for Nuclear Research, Dubna 141980, Russia; 3 Frumkin Institute of Physical Chemistry and Electrochemistry Russian Academy of Sciences, Moscow 119071, Russia

**Keywords:** microheterogeneous model, track-etched membrane, electrical conductivity, diffusion permeability, loose layer

## Abstract

The microheterogeneous model makes it possible to describe the main transport properties of ion-exchange membranes using a single set of input parameters. This paper describes an adaptation of the microheterogeneous model for describing the electrical conductivity and diffusion permeability of a track-etched membrane (TEM). Usually, the transport parameters of TEMs are evaluated assuming that ion transfer occurs through the solution filling the membrane pores, which are cylindrical and oriented normally to the membrane surface. The version of the microheterogeneous model developed in this paper takes into account the presence of a loose layer, which forms as an intermediate layer between the pore solution and the membrane bulk material during track etching. It is assumed that this layer can be considered as a “gel phase” in the framework of the microheterogeneous model due to the fixed hydroxyl and carboxyl groups, which imparts ion exchange properties to the loose layer. The qualitative and quantitative agreement between the calculated and experimental concentration dependencies of the conductivity and diffusion permeability is discussed. The role of the model input parameters is described in relation to the structural features of the membrane. In particular, the inclination of the pores relative to the surface and their narrowing in the middle part of the membrane can be important for their properties.

## 1. Introduction

Track-etched membrane (TEM) is a thin polymer film with a thickness in the range of 5–25 μm (usually from polyethylene terephthalate, polycarbonate or polyimide [1]) that was irradiated with heavy ions to form so-called «tracks», after which the material was etched under certain conditions with suitable chemicals. Etching determines the size and shape of the resulting pores [2]. This type of membranes has found a number of applications, most notably in the processes of micro- and ultra-filtration [3]. TEMs are widely used in medicine and biochemistry [4], in biological and chemical sensors [5], electrophoresis [6], optical sensors [7], and also as templates for the growth of hydrogels and nanowires of metals, semiconductors, and dielectrics [8]. The attractiveness of these membranes is due to the uniform size of pores of a regular shape, which is determined by the production method.

Indeed, the pores of TEMs have a strictly defined shape, and this shape can be different (e.g., cylindrical, conical, etc.) [9,10]. Note that the widespread idea about the cylindrical pore shapes of TEMs often is only an approximation, since the pores have a more complex geometry in reality. Thus, nanopores of polycarbonate membranes have rather a “barrel” shape [11]. TEM can have also a “cigar-like” [12,13] or a “toothpick” shape [14]. The shape of the pores of TEMs, as well as the final chemical composition of the surface, depend on many factors, such as: the chemical structure of the polymer, the conditions of irradiation and etching (temperature, time, exposure to UV irradiation, etching agents and surfactants) [15,16], as well as additional modification of the membranes (PVP coating or staining with dyes [17].

An important aspect in the context of this study is the fact that TEMs have a loose layer [18,19]) known also as a “loosened layer” [15] and “gel layer” [15,20,21]) on the pore wall [15,20,21], which appears as a result of the polymer damage by swift heavy ions and incomplete degradation of the polymer [15,22,23,24]. Since the etching takes place in an intermediate layer of a non-zero thickness [18,19], chain scission events may create end groups in the depth of a few nanometers. In the case of PET, these are –OH and –COOH groups. Therefore, this layer has ion-exchange properties and can swell in the presence of water and can be interpreted as an ion-exchange conducting gel layer [15]. At the same time, its hydraulic permeability should be negligible [20]. The thickness of the loose layer may vary depending on the type of polymer from which the membrane is made and the etching parameters. Dejardin et al. [15] estimated the thickness of this layer as approximately 1 nm based on the streaming potential measurements and based on accounting for the back current through the conductive loose layer. Based on the results of porosity values found from filtration data and data on the membrane electrical resistance reported by Sabbatovsky et al. [25], it is possible to evaluate the thickness of the loose layer in some PET TEMs as close to 2 nm; it is assumed in calculations that there is no convective flow in the loose layer, and the electrolyte electrical conductivity in this layer is equal to that in the bulk pore solution.

Modeling the transport properties and structure-property relationship of TEMs is an important task for predicting their behavior in various applications. Two main types of models serve as a theoretical basis. The first type of models, so-called «pore-flow» models [26], consider the membrane as a system of flow-through pores. They assume that the transport of particles is described inside a separate pore filled with a solution [27].

The second type of models, which is more common and is of the greatest interest for the present study, is called the “solution–diffusion” model [28]. Models of this type assume that the transported substances dissolve in the membrane material and then are transported through it under the action of a concentration gradient and/or a potential gradient. The general driving force in models of this type is the gradient of electrochemical potential. In turn, “solution–diffusion” models are divided into two groups. The first group includes the models that consider the membrane as a homogeneous medium [29]. The second group includes heterophase models [30], which consider the membrane as a heterogeneous system consisting of two (or more) phases, each of which is assigned with its own parameters.

In this paper, to describe the transport properties of a TEM, we apply the microheterogeneous model (MHM) [31], which is related to the second group. This model allows the description of transport characteristics of ion-exchange membranes, such as electrical conductivity, diffusion permeability, and ion transport numbers as functions of some structural and kinetic parameters [31,32]. The model is widely used by various laboratories and has shown its effectiveness in modeling the structure–property relationship for both commercial [33,34,35] and modified lab-made membranes [36,37,38,39]. For the first time, this model is applied to a TEM, where the ions may transport in aqueous pores of a specific geometry and a loose layer with ion-exchange properties. We also show that it is important to account for the fact that the pore axes are generally tilted at a certain angle to the membrane surface, which differs from 90°. We compare the results of modeling with experimental concentration dependencies of conductivity and diffusion permeability of a TEM. A part of model parameters is evaluated using the experimental values of the ion-exchange capacity, water content, and zeta potential for this membrane.

## 2. Theoretical

### 2.1. Basic Microheterogeneous Model (MHM)

The main idea of describing the ion transport in a multiphase membrane is to assign certain physicochemical properties to each domain (phase) and to describe the properties of the membrane as a whole as functions of the properties of individual domains, which is in line with the effective medium theory [40]. According to the basic MHM [31], the membrane under study consists of two nanophases. One of them is the gel phase formed by polymer chains with hydrated fixed and mobile ions, also including electrical double layers in the pore solution, which compensate the charge of the fixed ions so that the gel phase is electrically neutral. The second phase is an electroneutral solution that fills intergel spaces: the central parts of macro- and mesopores. The sum of the volume fractions of the gel ($f_{g}$) and the electroneutral solution ($f_{s}$) is equal to one ($f_{g}+f_{s}=1$). The model assumes that there is a local equilibrium between the gel phase and the electroneutral pore solution. The latter is identical to the external bulk solution [31].

It is convenient to introduce the conductance coefficients of ion *i* in the gel phase, $L_{i}^{g}$, and electroneutral solution, $L_{i}^{s}$, which are linked with the diffusion coefficients of ion i in the corresponding phases as gel, $D_{i}^{g}$, and solution $D_{i}^{s}$, respectively [31]:

(1) $$L_{i}^{g}=\frac{D_{i}^{g}c_{i}^{g}}{RT},\;L_{i}^{s}=\frac{D_{i}^{s}c_{i}^{s}}{RT},$$

where $c_{i}^{g}$ and $c_{i}^{s}$ are the ion concentrations in the respective phases; R is the gas constant; and T is the temperature.

The concentration of ions in the gel phase can be expressed through their concentration in the solution phase using the Donnan relation (written below for the case of a cation-exchange membrane) in a simplified form, valid for dilute solutions (<1 M) [41]:

(2) $$c_{+}^{g}c_{-}^{g}=K_{D}^{}c_{+}^{s}c_{-}^{s},$$

The electroneutrality condition in the gel phase and solution reads as:

(3) $$c_{+}^{g}=Q^{g}+c_{-}^{g},\;c_{+}^{s}=c_{-}^{s}=c,$$

where indexes “g” and “s” refer to the gel and electroneutral solution, respectively; $K_{D}^{}$ is the Donnan constant characterizing the interaction of coions with the matrix of the gel phase; the “+” and “−” indices refer to the counterion and coion, respectively (in case of a cation exchange membrane); $Q_{}^{g}$ is the ion-exchange capacity of the gel phase (concentration of charged fixed groups per unit volume of the gel). Equations (2) and (3) are written for the case of a 1:1 electrolyte, $z_{+}^{}=-z_{-}^{}=1$. Joint solution of these equations allows finding the cation and anion concentrations in the gel phase as functions of the electrolyte concentration in the intergel solution (the final equation is present in the Appendix A).

$Q_{}^{g}$ can be calculated when knowing the ion-exchange capacity of the membrane, $Q_{}^{mb}$, and the volume fraction of the gel phase, $f_{g}$:

(4) $$Q_{}^{g}=\frac{Q_{}^{mb}}{f_{g}},$$

When knowing $L_{i}^{k}$, it is possible to find the transport parameters in phase *k*, such as the ion transport number Equation (5), electrical conductivity Equation (6), and diffusion permeability Equation (7), according to the following relations [31,41]:

(5) $$t_{+}^{k}=\frac{L_{+}^{k}}{L_{+}^{k}+L_{-}^{k}},$$

(6) $$\kappa^{k}=(L_{+}^{k}+L_{-}^{k})F^{2}$$

(7) $$P_{}^{k}=2t_{+}^{k}L_{-}^{k}\frac{RT}{c}=\frac{(t_{+}^{k}L_{-}^{k}+t_{-}^{k}L_{+}^{k})RT}{c},$$

where $\kappa^{g}=(L_{+}^{g}+L_{-}^{g})F^{2}$ and $\kappa^{s}=(L_{+}^{s}+L_{-}^{s})F^{2}$ are the conductivity of the gel phase and electroneutral solution, respectively.

After calculating these parameters for each phase constituting the membrane, the corresponding parameters of the membrane as a whole, can be found using the following relations [42]:

(8) $$\kappa^{*}={\left(f_{g}{\left(\kappa_{}^{g}\right)}^{\alpha }+f_{s}{\left(\kappa_{}^{s}\right)}^{\alpha }\right)}^{1/\alpha }$$

(9) $$P_{}^{*}={\left(f_{g}{\left(P_{}^{g}\right)}^{\alpha }+f_{s}{\left(P_{}^{s}\right)}^{\alpha }\right)}^{1/\alpha }$$

where α is a structural parameter that characterizes the mutual arrangement of the membrane phases, −1≤α≤+1, where α=−1 corresponds to the in series arrangement, and α=1 corresponds to the parallel one.

When knowing $\kappa^{*}$ and $P_{}^{*}$, it is possible to calculate the ion transport numbers in the membrane using the following relation (deduced in the framework of the irreversible thermodynamics [43]):

(10) $$P_{}^{*}=\frac{2RT\kappa^{*}t_{+}^{*}t_{-}^{*}}{F^{2}c}$$

and the fact that

(11) $$t_{+}^{*}+t_{-}^{*}=1$$

Another way for reaching the membrane transport parameters is the use of a relation similar to Equations (8) and (9), but applied to the conductance coefficient of each ion [31]:

(12) $$L_{i}^{*}={\left(f_{g}{\left(L_{i}^{g}\right)}_{}^{\alpha }+f_{s}{\left(L_{i}^{s}\right)}_{}^{\alpha }\right)}^{1/\alpha },$$

After calculating the effective membrane transport coefficients, the values of $\kappa^{*}$, $P_{}^{*}$, and $t_{+}^{*}$ can be found using Equations (5)–(7), in which superscript “*k*” is replaced with “*” [31].

Both ways for calculating the effective transport parameters of heterogeneous medium use similar equations, Equations (8) and (9) in the first case, and Equation (12) in the second case. The above equations (Equations (8) and (9), on one hand, and Equation (12), on the other) are different ways of generalization of the two limiting situations, when the conducting phases are arranged in series or in parallel. In both cases, it is necessary to know the following six parameters determining the transport properties of a specific membrane: two thermodynamics coefficients, *K_D_* and $Q^{g}$; two structural parameters, *f_g_* and *α*; and two kinetic ones, $D_{+}^{g}$ and $D_{-}^{g}$. The diffusion coefficients in the inter-gel solution, $D_{i}^{s}$, are assumed to be the same as in free solution (tabular values). If these parameters are known and the external electrolyte concentration is set, it is possible to calculate, first, the ion concentrations in the gel phase, Equations (2) and (3); then, the $L_{i}^{g}$ and $L_{i}^{s}$ coefficients, from Equation (1), are considered. After that, when using the first way, we calculate first the conductivities and diffusion permeabilities of different phases by using Equations (5)–(7), and then we determine the conductivity and diffusion permeability of the membrane by using Equations (8) and (9); finally, the transport numbers are calculated using Equations (10) and (11) (the equations are present in the Appendix A). When using the second way, $L_{i}^{*}$ effective coefficients are calculated via Equation (12), and then the membrane transport parameters are found, as explained in the comment to Equation (12).

In this study, we report the results obtained using the first way, i.e., Equations (5)–(10), since the agreement between the calculations and experiment was better than when using the second way.

### 2.2. Problem Formulation

**Figure 1:**
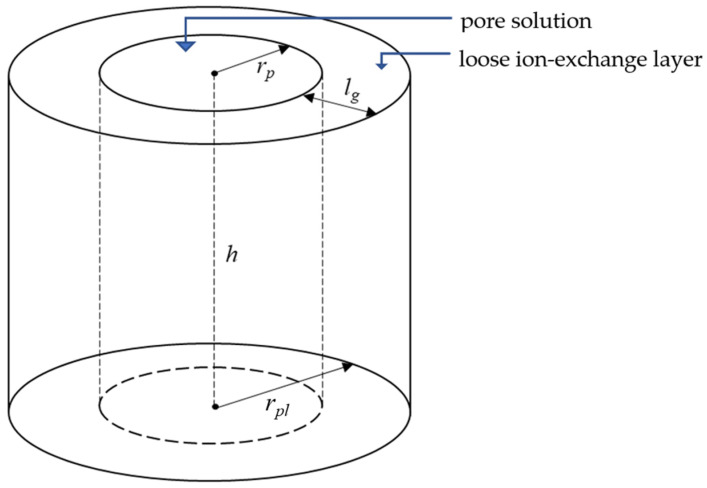
Schematic representation of a system that includes a solution in a TEM pore with a radius *r_p_* and a loose (gel) layer with a thickness of *l_g_*.

When describing the properties of a TEM, Sarapulova et al. [34] assumed that the transfer occurred only through a solution that fills the membrane pores. In this study, we take into account an additional way for ion transfer: an intermediate loose layer between the pore solution and membrane bulk; the latter is a dense material not permeable for ions and water. Figure 1 shows a schematic representation of a pore and loose layer in a TEM, assuming that the pore has a cylindrical shape.

The volume of the solution in one pore of the membrane is equal to:

(13) $$V_{s}=\pi r_{p}^{2}h,$$

where *h* is the cylinder height (membrane thickness).

The loose conductive layer will be considered as a “gel phase” within the framework of the MHM. We assign to this phase the diffusion coefficients of cations, $D_{+}^{g}$, and anions, $D_{-}^{g}$, as well as the Donnan constant, $K_{D}^{}$, in order to take into account the contribution of this layer. This will make it possible to calculate the ion concentrations in this phase using Equations (2) and (3), and then we can calculate the conductivity, $\kappa_{}^{g}$, and diffusion permeability, $P_{dif}^{g}$, using Equations (5)–(7). Similarly, the values of $\kappa_{}^{s}$ and $P_{}^{s}$ can be found using the same equations with $L_{i}^{s}$ coefficients.

The radius and volume of the “pore solution + loose layer” system (abbreviated as “pore + layer”) are found as:

(14) $$r_{pl}=r_{p}+l_{g},$$

(15) $$V_{pl}=\pi {\left(r_{p}+l_{g}\right)}^{2}h,$$

where $l_{g}$ is the thickness of the loose layer.

The volume fraction of the solution in the “pore + layer” system, which means the $f_{s}^{}$ parameter when applying the MHM to this system, is equal to:

(16) $$f_{s}^{}=\frac{V_{s}}{V_{pl}}={\left(\frac{r_{p}}{r_{p}+l_{g}}\right)}^{2},$$

Note that, even if the pore axis is not perpendicular to the membrane surface, Equation (16) remains correct. The volume fraction of the “gel phase” (loose layer) in this system is equal to:

(17) $$f_{g}^{}=1-f_{s}^{},$$

Now the calculation of the conductivity, $\kappa^{*}$, and diffusion permeability, $P^{*}$, of the “pore + layer” system is possible, when using Equations (8) and (9), respectively. In addition, the ion transport numbers in this system, $t_{i}^{*}$, can be found using Equation (10). Since $t_{i}^{*}$ is a relative dimensionless quantity, it characterizes the TEM as a whole: $t_{i}^{mb}$ = $t_{i}^{*}$. However, to find the conductivity, $\kappa^{mb}$, and diffusion permeability of the membrane, we have to take into account that it contains, together with pores, the hydrophobic material not acceptable to water and ions.

To find the value of conductivity for the membrane as a whole, $\kappa^{mb}$ (as a property of the material), we find the conductance of one pore together with its loose layer (as a property of the component), $\kappa_{pl}=\kappa^{*}\pi {\left(r_{p}+l_{g}\right)}^{2}/h$, and then we determine the conductance of the membrane with an area of 1 cm^2^, where $n_{p}$ is the number of pores per 1 cm^2^ of surface. The value $\kappa^{mb}$ is further defined as $\kappa^{mb}=\kappa^{pl}h$. Finally:

(18) $$\kappa^{mb}=\kappa^{*}\pi {\left(r_{p}+l_{g}\right)}^{2}n_{p}={\left(f_{g}{\left(\kappa_{}^{g}\right)}^{\alpha }+f_{s}{\left(\kappa_{}^{s}\right)}^{\alpha }\right)}^{1/\alpha }\pi {\left(r_{p}+l_{g}\right)}^{2}n_{p},$$

Similarly, for the (differential) diffusion permeability of the membrane, $P^{mb}$ is:

(19) $$P^{mb}=P^{*}\pi {\left(r_{p}+l_{g}\right)}^{2}n_{p}={\left(f_{g}{\left(P_{}^{g}\right)}^{\alpha }+f_{s}{\left(P_{}^{s}\right)}^{\alpha }\right)}^{1/\alpha }\pi {\left(r_{p}+l_{g}\right)}^{2}n_{p},$$

## 3. Experimental

### 3.1. Membrane

In the current study, a TEM obtained at the Flerov Laboratory of Nuclear Reactions, Joint Institute for Nuclear Research (FLNR JINR) (Dubna) is considered. The membrane under study (with the provisional name TEM#811) is made of polyethylene terephthalate (PET) film (manufactured by Vladimirskii khimicheskii zavod, Russia). The main characteristics of TEM#811 are presented in Table 1.

**Figure 2:**
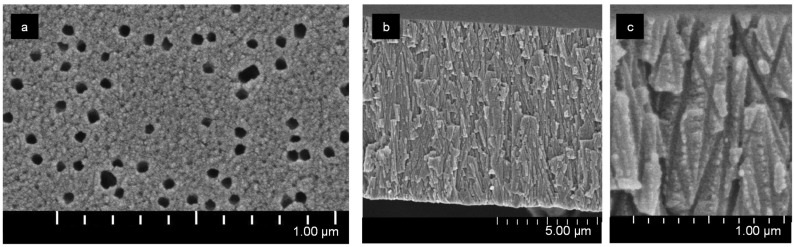
SEM images of surface (**a**) and cross section (**b**,**c**) of TEM#811.

Figure 2 represents scanning electron microscopy (SEM) images of the membrane surface. Figure 2a shows a rough surface structure with a characteristic scale of about 10 nm or less.

During the manufacture of TEM#811, hydroxyl and carboxyl groups are formed on the polymer surface after etching the tracks, which determine the negative electrical charge of the surface [1,34,45]. The pores of TEM#811 have a shape close to cylindrical, although there are reasons to believe that the shape of the pores is more similar to an elongated double cone («hourglass») [34]. This assumption is supported by the pore radius values, *r_p_*, determined from SEM images (Figure 2a) (20 nm) [44], and estimated by hydraulic permeability (14 nm) [44].

### 3.2. Solution

In the experiments, we used a solution prepared from solid NaCl of analytical grade (JSC “Vekton”). Deionized water with the electrical conductivity of 0.5 μS cm^−1^ and pH of 5.50 ± 0.05 was used.

### 3.3. Measurement of Transport Characteristics

In this study, the pretreatment of TEM was carried out similarly to the pretreatment of ion exchange membranes [46]. All samples were successively exposed for 24 h in NaCl solutions, the concentration of which was 300 and 30 g dm^−3^. All experiments were carried out at 25 ± 1 °C.

The specific electrical conductivity of the membranes, $\kappa^{mb}$, was determined by a differential method using a pince-like cell [47,48] and a MOTECH MT4080 immittance meter (Motech Industries Inc. Taiwan) at an alternating current frequency of 1 kHz. The calculation of $\kappa^{mb}$ was performed by the following equation:

(20) $$\kappa^{mb}=\frac{h}{R_{mb+s}-R_{s}}$$

where *h* is the membrane thickness; $R_{mb+s}$, measured resistance of the membrane and solution; $R_{s}$, resistance of solution.

The diffusion permeability was measured using a flow-type two-chamber cell. The membrane under study separated two ducts. Distilled water was pumped through one of them (duct 1), while a salt solution with a given concentration was pumped through the other (duct 2). The scheme of the cell, procedure for experiment and processing of the data obtained are described in detail in [32]. The following equation was used to calculate the integral coefficient of diffusion permeability, P:

(21) $$P=j\frac{h}{C^{2}}=\frac{d(C^{1}V^{1})}{dt}\frac{h}{S_{mb}C^{2}}$$

where j is the density of the salt diffusion flux through the membrane; $S_{mb}$, area of the membrane; $C^{1}$ и $C^{2}$, salt concentrations in ducts 1 and 2, respectively; $d(C^{1}V^{1})/dt$ is the rate of the increase in the salt concentration in duct 1; $V^{1}$ is the volume of the solution in this duct.

Differential diffusion permeability coefficient, P*, was determined from the concentration dependence of *P* using the following equation [31]:

(22) $$P^{*}=P\left(1+\frac{d\ln P}{d\ln c}\right)$$

### 3.4. Ion-Exchange Capacity

Total ion-exchange capacity (*Q^mb^*) of weakly ionized membranes is determined by a static method [49]. The membrane samples, the weight of which in dry state (*m_dry_*) was about 0.5 g, were preliminarily transformed into the H^+^ form, washed in distilled water, ground, and placed into conical flasks. 20.00 cm^3^ of 0.01 mol dm^−3^ sodium acetate solution was added with a pipette and kept for 24 h, shaking occasionally. The potentiometric titration of this solution (with membranes placed into it) was carried out with a 0.01 mol dm^−3^ NaOH solution using an EasyPlusTitrators autotitrator (METTLER TOLEDO). More details are reported in [34].

### 3.5. Zeta Potential

Streaming potential (Δ*E*) measurements were performed in the dead-end filtration mode using a cylindrical cell made of polymethyl methacrylate 250 mm long and 38 mm in inner diameter with two silver chloride electrodes located above and below the membrane and a tap for supplying compressed nitrogen, creating a pressure drop (Δ*P*) over the membrane. After mounting the membrane in the lower part of the cell, an electrolyte solution was poured through the upper hole, and the upper hole was sealed, and compressed nitrogen was supplied. During the measurements, a pressure drop of up to 2.5 bar was used. A high resistance voltmeter was used to record the values of streaming potential. The value of specific electrical conductivity of the solution, $\kappa_{0}$, was separately measured in each case in a conductometric cell with blackened platinum Pt/PtO_2_ electrodes. The ζ potential was calculated by the Helmholtz–Smoluchowski equation:

(23) $$\zeta =\left(\Delta E/\Delta P\right)\kappa_{0}\eta /\varepsilon \varepsilon_{0}$$

where ε and $\varepsilon_{0}$ are the relative and vacuum permittivity, respectively; *η* is the viscosity of the solution.

The value of surface charge, *σ*, is estimated using the Grahame equation [50]:

(24) $$\sigma =\sqrt{8\varepsilon \varepsilon_{0}cRT}\times \sinh\left(\frac{\zeta F}{2RT}\right)$$

## 4. Results and Discussion

### 4.1. Model Input Parameters

All parameters of the simulation are presented in Table 2. The values of some of the parameters were estimated based on the following consideration.

Ion-exchange capacity of membrane, *Q*^mb^ (in mol/L of membrane), measured as described in Section 3.4, is total. It includes the contribution of all ionogenic groups that are deprotonated at high pH of the external solution. As mentioned above, the fixed ionogenic groups in PET membranes are –OH and –COOH. For the terephthalic acid, containing COOH groups similar as the PET matrix, pK_a1_ = 3.54 [23], hence, at pH = 5.5 (at which the conductivity measurements were made), most of these groups should be dissociated. However, it is known that the conductivity of PET TEMs increases in the pH range from 4 to 7, which indicates that the degree of dissociation increases in this range of pH [25]. Apel et al. [23] explain this result by the fact that the degree of dissociation of functional groups in constrained space, in particular, in nanopores in PET, can significantly decrease compared to the bulk value [53]. Chen and McCarthy [54] estimate the content of COOH and OH groups in the surface layer (after alkaline etching) as approximately 0.06 and 0.1 groups per 1 repeating unit of PET. Therefore, the fraction of the COOH groups among all functional groups is 6/16. Taking into account that hydroxyl groups do not dissociate at pH = 5.5, we considered it reasonable to assume that, at this pH value, the fraction of the dissociated groups is 0.25 of the total functional groups (0.064 M) and is equal to 0.015 M (per L of the swollen membrane) (Table 2). The ion-exchange capacity of the loose gel layer, that is, the concentration of charged fixed groups in this layer, $Q^{g}$ (in mol/L of loose layer) in accordance with Equation (4), will be equal to:

(25) $$Q^{g}=Q^{mb}/f_{g}^{mb}=Q^{mb}/(f_{p}f_{g}^{})$$

where $f_{p}=\pi {\left(r_{p}+l_{g}\right)}^{2}n_{p}$ is the fraction of the membrane surface occupied by the “pore + layer” systems.

The value of $Q^{g}$ can also be estimated from the data on the cross-streaming potential in the membrane pores obtained in the dead-end filtration mode (Section 3.5). The zeta-potential of the pore surface is ζ = −10 mV in a 0.01 M KCl solution. This value is approximately three times less than in the case of a homogeneous CMX membrane, for which ζ = −28 mV in a 0.02 M NaCl solution [55]. The estimations of the surface charge density, *σ*, using the Grahame equation, Equation (24), give 0.2 and 0.9 μC/cm^2^ for the pore walls of the TEM#811 and for the CMX membrane, respectively. The result for the TEM#811 correlates with the value of 0.35 μC/cm^2^ reported by Sabbatovskiy et al. [25], for a PET TEM with a radius 17 nm. Chen and McCarthy [54] estimated the surface concentration of COOH on etched PET surface as 2.5 × 10^13^ 1/cm^2^, which gives *σ* ≈ 0.4 μC/cm^2^ when all these groups are dissociated.

The ion-exchange capacity of the CMX membrane is 1.6 M [56,57,58], and the gel fraction is approximately equal to 0.85–0.9 [56,58]. Hence, it follows that the ion-exchange capacity of the gel phase in the CMX membrane is approximately equal to 2 M. Assuming a linear dependence of the surface charge on its volume value, the ion-exchange capacity of loose layer at pH = 5.5 can be defined as $Q^{g}$ = 0.45 M.

**Figure 3:**
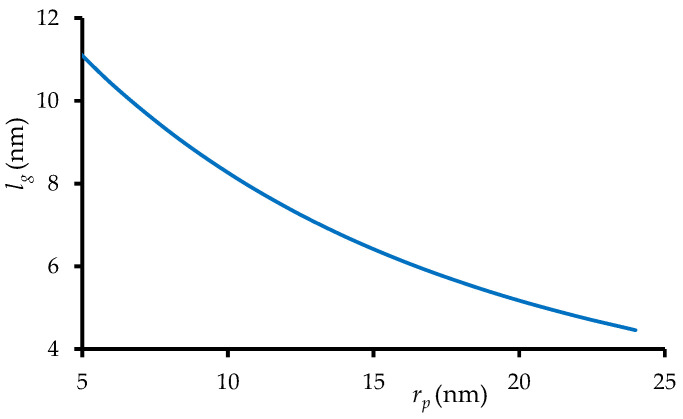
The thickness of loose layer as a function of pore radius found using Equation (26) with *n_p_* = 5.0 × 10^9^ pores/cm^2^, *Q^g^* and *Q^mb^* are indicated in Table 2.

Knowing $Q^{g}$ and $Q^{mb}$ values, it is possible to estimate the thickness of the loose layer («gel phase»), *l*_g_. Indeed, the values of *l*_g_ and *r*_p_ define $f_{p}$ and $f_{g}^{}$ values in Equation (25), considering that $n_{p}$ is known with sufficient accuracy from SEM images. It is possible to find the dependence of *l*_g_ on *r*_p_ (Figure 3) using Equation (26), which is obtained by substituting Equation (4) into (17) (taking into account Equation (16)) and then solving the quadratic equation:

(26) $$l_{g}=\frac{Q^{mb}}{\pi n_{p}Q^{g}\left(r_{p}+\sqrt{r_{p}^{2}+Q^{mb}/\pi n_{p}Q^{g}}\right)}$$

Since $Q^{g}$ и $Q^{mb}$ are fixed, the global volume fraction of the charged porous material in the loose layer, $f_{g}^{mb}$, does not change when varying *r*_p_. Therefore, *l*_g_ increases with decreasing *r*_p_. Note that the calculated values of *l*_g_ become larger than the pore radius if *r*_p_ < 10 nm (Figure 3).

The loose layer is assumed to be a porous polymer (PET) material, which is the result of incomplete etching. Apparently, it looks similar to a non-crosslinked ion-exchange gel with larger pores than in homogeneous ion-exchange membranes. Therefore, it can be expected that the ion diffusion coefficients in this layer are significantly greater than in homogeneous ion-exchange membranes, but lower than in free solution. We assume that $D_{+}^{g}$ is several times less than in free solution, however, $D_{-}^{g}$ is about one order of magnitude less than $D_{+}^{g}$. The latter is due to the fact that, in a porous medium, there are some narrow pores with charged walls, which are easily permeable to a counterion, but impermeable to a coion. As a result, the path for the coion is much more tortuous than for the counterion. However, $D_{+}^{g}$ and $D_{-}^{g}$ are fitting parameters in the model. To find them, we take into account that the gel conductivity is very sensitive to the value of $D_{+}^{g}$ (and almost does not depend on $D_{-}^{g}$). On the contrary, the diffusion permeability strongly depends on $D_{-}^{g}$, but is not sensitive to $D_{+}^{g}$.

The characteristic value of the Donnan equilibrium constant can be estimated from the consideration presented in our paper [59]. When the concentrations are expressed in mol per m^3^ of free water within a membrane pore, the Donnan constant should be of the order of 1. Additionally, when ion concentrations are expressed in mol per m^3^ of swollen membrane, the free water content should be taken into account. The latter is about 20–30 vol.% in gel ion-exchange membranes and expected to be close to 50 vol.% in the loose layer. Then, the *K*_D_ value should be of the order of 0.1 at water content equal to 30 vol.(%). In our calculations, we used the value of *K*_D_ = 0.2.

The membrane pore radius, *r*_p_, was previously determined [44] by two independent methods: using SEM images (which gives 20 nm) and by using hydraulic permeability (14 nm). This difference is most likely due to the hourglass shape of the pores: the radius is larger on the membrane surface and smaller in the volume. The hourglass shape of the pores is due to the etching stage. The pore radius is more than two orders of magnitude smaller than its length; the delivery of a chemical reagent that etches the membrane material is carried out mainly due to its diffusion. As a result, the etching process along the length of the pore proceeds at different rates: the maximum rate is at the entrance to the pore and decreases towards its middle. We have carried out calculations for a wide range of pore radius, including both of the above values.

### 4.2. Modelling of Transport Characteristics for Cylindrical Pores Perpendicular to the Surface

When calculating membrane transport characteristics by applying the MHM, we first assume that pores in the TEM#811 are cylindrical and their axes are perpendicular to the surface. Then, we set *α* = 1 in Equations (8) and (9). In calculations, the entire solution filling the pore outside the loose layer is assumed to be electrically neutral. We use this assumption, taking into account that, in the studied concentration range (0.1–0.5 M), the pore radius of the TEM#811 is almost an order of magnitude greater than the Debye length. Double electrical layers in the pores of such a membrane do not overlap, and a relatively high permselectivity is found in refs. [44,60], which is unattainable due to the formation of these layers in the pores. Thus, for a quantitative description of the characteristics of this membrane, it is necessary to use the MHM described above.

**Figure 4:**
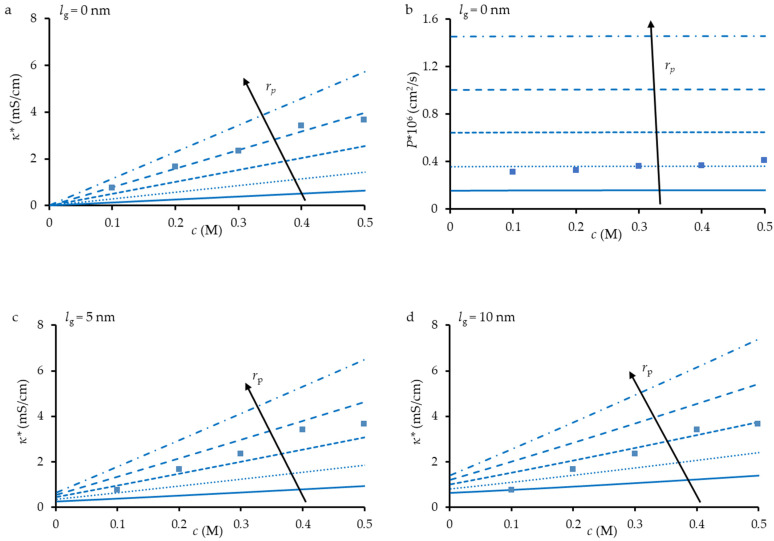

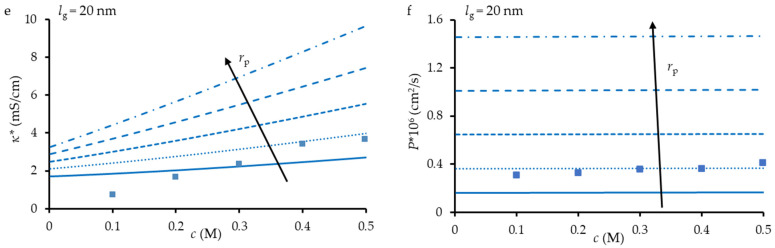
Experimental (dots) and simulated (lines) results for the electrical conductivity (**a**,**c**,**d**,**e**) and diffusion permeability (**b**,**f**) of TEM#811 as a functions of NaCl concentration. Simulations are made for *r*_p_ = 8, 12, 16, 20 and 24 nm at *l*_g_ = 0 (**a**,**b**), 5 nm (**c**), 10 nm (**d**), and 20 nm (**e**).

Figure 4 show the simulated concentration dependencies of the conductivity and diffusion permeability of the TEM#811 found at different pore radius and different thicknesses of loose layer, *l_g_*. The other input parameters are indicated in Table 2.

An increase in the thickness of the loose layer leads in increasing the membrane conductivity but has no effect on its diffusion permeability. We give the P* vs. *c* dependence only in the case of *l_g_* = 0 and *l_g_* = 20 nm (for comparison) because the eye does not notice the difference in the course of such curves at other values of *l_g_*. This is explained by the fact that membrane diffusion permeability is controlled by the transport of coions, Cl^−^ ion in our case. Since the coions are excluded from the loose layer (Donnan exclusion [49]), the Cl^−^ ion concentration there is significantly lower than in the external equilibrium solution. In addition, the Cl^−^ diffusion coefficient in this layer is much less than in free solution. As a consequence, the diffusion flux through the loosed layer, in the first approximation, is equal to $D_{-}^{g}c_{-}^{g}l_{g}/h$, which is much lower the diffusion flux through the pore solution, proportional to $D_{-}^{s}c_{-}^{s}r_{p}/h$. As for the membrane conductivity, the cation-exchange loose layer gives an important contribution to the value of this parameter due to high counterion (Na^+^ ion) concentration in this layer: $\kappa^{g}=(D_{+}^{g}c_{+}^{g}+D_{-}^{g}c_{-}^{g})F^{2}/RT$, especially at low concentrations in the external solution since, in this concentration range, $c_{+}^{g}>>c_{+}^{s}$. Besides, as we noted above, $D_{+}^{g}>D_{-}^{g}$.

It can be seen that, if we take *l_g_* = 0, the *κ^∗^* vs. *c* and P* vs. *c* dependencies can be described quantitively. However, the first dependence is well described at *r*_p_ = 20 nm, and the second one is at *r*_p_ = 12 nm. Simultaneously, both dependences with one set of parameters cannot be described, even if we change the *l_g_* value. This discrepancy can be explained by two factors.

First, this can be linked with the deviation of the shape of the pore from cylindrical. Note that the value of *r*_p_ fitting the *κ^∗^* vs. *c* dependence is higher than the hydraulic pore radius; the value of *r*_p_ fitting the P* vs. *c* dependence is less than the hydraulic pore radius. If we assume that the pore is conical, as in an hourglass, we can arrive at a higher conductivity at a lower pore radius due to the cation (counterion) conductance of the loose layer. At the same time, the cation-exchange loose layer does not contribute to the anion permeation since coions are excluded from a cation-exchange material. Therefore, the narrow part of the pore can assure a low diffusion permeability detected experimentally.

The second factor could be the intersection of pores within the membrane when they are not perpendicular to the surface (Figure 4c). At such an intersection, there appears a path when ions cross the interface between the loose layer and pore solution, i.e., the conductive phases can be arranged in series. This arrangement does not affect significantly the membrane conductivity (since the loose layer has a good counterion conductance), but it essentially reduces the diffusion permeability (the loose layer is low permeable for coions).

Note that series arrangement takes place if the pores are hourglass shaped, even when the pore axis is perpendicular to the surface.

### 4.3. Application of the MHM with α < 1

As written above, two factors can explain the failure of the assumption that the pores are cylindrical and perpendicular to the membrane surface studied in Section 4.2: (1) the hourglass-like shape of the pores and (2) the intersection of inclined pores within the membrane. In both cases, there appear in series arrangement of two conductive phases, which can be modelled in the frame of the MHM by setting *α* < 1.

**Figure 5:**
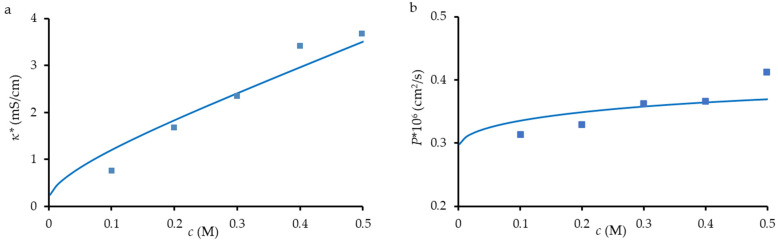
Experimental (dots) and simulated (lines) electrical conductivity (**a**) and diffusion permeability (**b**) of TEM#811 as functions of NaCl concentration. Simulations were made using the parameters presented in Table 2.

Figure 5 shows the results of such a simulation performed with a reasonable value of *α* = 0.34. Note that for ion-exchange membranes, the value of *α* is mainly in the range 0.2–0.3 [31,61]. The value of *r*_p_ = 18 nm is also reasonable, being lower than that determined by the SEM images and higher than that from hydraulic permeability measurements. The other parameters are indicated in Table 2. The ideal agreement between the simulation and experiment is not achieved, but the tendency for the increase in electrical conductivity and diffusion permeability is well observed.

**Figure 6:**
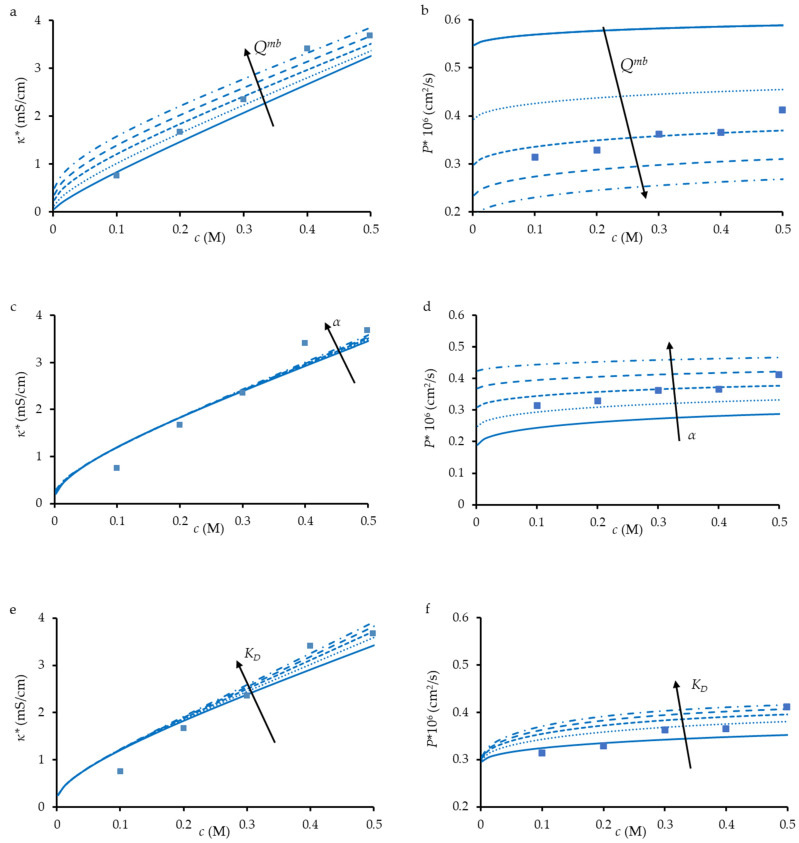
Experimental (dots) and simulated (lines) electrical conductivity (**a**,**c**,**e**) and diffusion permeability (**b**,**d**,**f**) of TEM#811 as functions of NaCl concentration. Simulations were performed using the parameters presented in Table 2. In each figure, one parameter was varied, while others were fixed: *Q*^mb^ = 0.005, 0.010…0.025 M (**a**,**b**), α = 0.25, 0.3 … 0.5 (**c**,**d**), and *K*_D_ = 0.1, 0.3 … 0.9 (**e**,**f**).

Figure 6 show the effect of different parameters on the course of κ* vs. *c* and P* vs. *c* dependencies. As can be seen, the membrane ion-exchange capacity, *Q^mb^*, affects both κ * than P*. However, an increase in *Q^mb^* leads to a growth in κ *, but to a decrease in P*. As mentioned above, the conductivity of the loose layer is proportional to the concentration of cations in it, which is proportional to *Q^mb^* in the first approximation. On the contrary, an increase in *Q^mb^* strengthens the Donnan exclusion of coions, which reduces the electrolyte diffusion in the loose layer.

Parameter *α* has practically no effect on κ*: the conductivities of the pore solution and the loose layer are comparable, hence, it does not matter how the phases are located relative to each other. However, the diffusion permeability of the loose layer is much lower than that of the pore solution. For this reason, P* decreases rapidly as *α* decreases: the smaller *α* is, the more and more coions have to cross the ion-exchange material in order to move from one side of the membrane to the other.

As for *K*_D_, which determines (together with *Q^g^*) the coion concentration in the loose layer, its increase almost does not affect the membrane conductivity, since the contribution of coions in this layer is quite low. However, the effect of *K*_D_ on the diffusion permeability is significant: an increase in *K*_D_ leads to an important increase in the coion concentration in the loose layer, which results in a higher P*.

The comparison between the experimental and simulated results (Figure 5) shows a qualitative agreement between them. However, this agreement could perhaps be better if one takes into account the expected hourglass-like shape of the pore and the presence of an electrical double layer (EDL) on the pore walls. We have discussed above how this shape could improve the model fidelity, mainly by introducing an in-series connection of the ion-exchange material and the pore solution. As for the EDL, its role indeed is insignificant when the pore radius is close to 18 nm, as it was used in the calculations of the curves in Figure 5. However, in the case of hourglass-like pore shape, it can be imagined that the pore radius in its narrowest part can be significantly lower, and then the EDL thickness can be comparable with the pore radius.

Interestingly, different methods give different pore radius (Table 2). These deviations can be explained by taking into account the peculiarities of each method and the fact that, in all cases, the pores are assumed to be cylindrical. Indeed, the lower value of *r_p_* (14 nm) obtained from hydraulic permeability compared to that found from SEM images (20 nm) should be due to narrowing of the pores in the middle part of the membrane (if it is expected to have an hourglass-like shape). The value of *r_p_*, found by fitting the parameters of the MHM, is 18 nm, i.e., it lies in a fork between two mentioned above values. This is logical since *r_p_* = 18 nm is a trade-off when modeling: a higher value of *r_p_* allows a better fit when describing the conductivity, while a lower value is better for describing the diffusion permeability. As for 22 nm, estimated from the difference between the density of the dry TEM#811 and the PET foil density, this value apparently reflects the porous structure of the loose layer: although this layer is not hydraulically permeable, it has pores which reduce the measured density of the dry membrane. If the porosity of the loose layer is set equal to 50% (as assumed in Section 4.1 when evaluating *K*_D_), then, to obtain the density of TEM#811 equal to 1.30 g cm^−3^, it is necessary to set *r_p_* = 18.8 nm to obtain, by calculation, the TEM#811 density equal to 1.30 g cm^−3^; the PET density is 1.41 g cm^−3^ (Table 1).

## 5. Conclusions

We have shown that, when assuming that the pores in a TEM are cylindrical and their axes are perpendicular to the membrane surface, it is impossible to describe simultaneously the membrane conductivity and diffusion permeability by using one set of input parameters. To fit the conductivity, the pore radius should be relatively great, and to fit the diffusion permeability, it should be small. When assuming that the pores are hourglass shaped and/or that the pores are inclined to the surface and can intersect within the membrane, we can obtain a qualitative agreement between the experimental and simulated data using the MHM. The model allows accounting for the presence of an in-series connection of the pore solution and ion-exchange material of the loosed layer by introducing a structural parameter *α* < 1. However, we believe that the quantitative agreement could perhaps be better if one assume the hourglass-like shape of the pores and takes into account presence of an electrical double layer (EDL) on the pore walls.

## Figures and Tables

**Table 1:** Some characteristics of TEM#811.

Thickness *	Pore Density *, *n_p_*		**Pore Radius, *r_p_***		**Surface Porosity**		**Fixed Groups [17]**
10 μm	5.0 × 10^9^ pores/cm^2^		20 nm * 14 nm ** 22 nm ***		0.063		hydroxyl and carboxyl groups
**Water Uptake** [34]		**Hydraulic Permeability** [44]		**Density (Dry)** [44]		**Total Ion-Exchange Capacity, *Q_tot_^mb^*** [34]	
5%		5.0 × 10^−3^ cm^3^/(cm^2^ min)		1.30 ± 0.02 g cm^−3^		0.064 ± 0.003 mmol g^−1^ wet	

* Estimated by SEM. ** Estimated by hydraulic permeability. *** Estimated from the difference between the density of the dry TEM#811 and the PET foil density, 1.41 ± 0.01 g cm^−3^.

**Table 2:** Input parameters used in the calculation of the conductivity and diffusion permeability of the membrane.

Parameter	Value	Description	Source
*α*	0.34	Structural parameter	*
*c*	0.1–0.5 M	Concentration of free solution	**
$D_{+}^{s}$	1.33 × 10^−9^ m^2^/s	Ion diffusion coefficients in the free solution	[51,52]
$D_{-}^{s}$	2.04 × 10^−9^ m^2^/s		
$D_{+}^{g}$	9 × 10^−10^ m^2^/s	Ion diffusion coefficients in the membrane	*
$D_{-}^{g}$	0.1 × 10^−10^ m^2^/s		*
*K_D_*	0.2	Donnan’s constant	*
*n_p_*	5 × 10^9^ 1/cm^2^	Density of pores on the surface	**
*Q^mb^*	0.015 mol/L of the swollen membrane	Concentration of fixed charged groups at pH = 5.5	**
*Q^g^*	0.450 mol/L of the loose layer	Ion-exchange capacity of the loose layer of the membrane	**
*r_p_*	18 nm	Pore radius	*
*l_g_*	5.6 nm	Thickness of the loose layer	*
*T*	298.15 K	Temperature of the system	**

* fitting parameter. ** measured value.

## Data Availability

Not applicable.

## Appendix A

The co-ion (anion in our work) concentration in the gel phase and transport number of counterions in the membrane as functions of the electrolyte concentration in the intergel solution may be found using the following equations:

(A1) $$c_{-}^{g}=-\frac{Q^{g}}{2}+\sqrt{{\left(\frac{Q^{g}}{2}\right)}^{2}+K_{D}^{}c^{2}}$$

and

(A2) $$t_{+}^{*}=\frac{1}{2}+\sqrt{\frac{1}{4}-\frac{P_{}^{*}F^{2}c}{2RT\kappa^{*}}}$$
